# Estimating ground reaction force with novel carbon nanotube-based textile insole pressure sensors

**DOI:** 10.1017/wtc.2023.2

**Published:** 2023-03-02

**Authors:** Kaleb Burch, Sagar Doshi, Amit Chaudhari, Erik Thostenson, Jill Higginson

**Affiliations:** Department of Mechanical Engineering, University of Delaware, Newark, DE, USA

**Keywords:** sensors, monitors, embedded electronics, biomechanics, biomechatronics

## Abstract

This study presents a new wearable insole pressure sensor (IPS), composed of fabric coated in a carbon nanotube-based composite thin film, and validates its use for quantifying ground reaction forces (GRFs) during human walking. Healthy young adults (*n* = 7) walked on a treadmill at three different speeds while data were recorded simultaneously from the IPS and a force plate (FP). The IPS was compared against the FP by evaluating differences between the two instruments under two different assessments: (1) comparing the two peak forces at weight acceptance and push-off (2PK) and (2) comparing the absolute maximum (MAX) of each gait cycle. Agreement between the two systems was evaluated using the Bland–Altman method. For the 2PK assessment, the group mean of differences (MoD) was −1.3 ± 4.3% body weight (BW) and the distance between the MoD and the limits of agreement (2S) was 25.4 ± 11.1% BW. For the MAX assessment, the average MoD across subjects was 1.9 ± 3.0% BW, and 2S was 15.8 ± 9.3% BW. The results of this study show that this sensor technology can be used to obtain accurate measurements of peak walking forces with a basic calibration and consequently open new opportunities to monitor GRF outside of the laboratory.

## 1. Introduction

There has been an increasing interest in the use of wearable sensors to quantify human gait (Tao et al., 2012; Muro-de-la-Herran et al., 2014; Chen et al., 2016) for applications such as monitoring of partial weight-bearing (PWB) rehabilitation (van Lieshout et al., 2016), control of exoskeletons (Bae et al., 2018), and analysis of sport performance (Gouwanda and Senanayake, 2008; Raper et al., 2018). However, it is difficult to reproduce the accuracy and reliability of standard lab equipment, such as motion capture systems and force plates (FPs), outside of a laboratory setting (Chen et al., 2016). FPs are used to measure ground reaction force (GRF) for gait research and clinical applications, such as monitoring maximum forces for patients prescribed with a PWB regimen. PWB specifically seeks to limit maximum GRF during gait, which makes maximum GRF a useful clinical metric.

In particular, the goal of PWB is often to restrict peak GRF, and a sensor that exhibits a lower limit of agreement (as estimated using the Bland–Altman method) which lies within the prescribed range of peak forces during PWB has been proposed as a suitable criterion for biofeedback applications (van Lieshout et al., 2016). Although FPs easily meet this criterion, they are costly (USD$ 21,000–36,000 (Muro-de-la-Herran et al., 2014) and can only measure forces at a fixed location, which makes them impractical for obtaining walking forces outside of a laboratory. Data collection with FPs requires a trained professional to conduct a motion analysis study and lengthy setup time, both of which limit the ability to collect large datasets (Simon, 2004).

Alternatively, GRF can be measured portably with an insole pressure sensor (IPS) (Bamberg et al., 2008; Fong et al., 2008; Rouhani et al., 2010; Dyer and Bamberg, 2011; Howell et al., 2013; Muro-de-la-Herran et al., 2014; Braun et al., 2015; Jacobs and Ferris, 2015; Chen et al., 2016; Seiberl et al., 2018). Slight differences exist in measuring with an IPS versus a FP, where a FP can often read force in three dimensions and the sensor can only read force normal to the plantar surface of the foot. This discrepancy is often handled by calibrating IPSs to vertical GRF (Bamberg et al., 2008; Fong et al., 2008; Rouhani et al., 2010; Dyer and Bamberg, 2011; Braun et al., 2015; Seiberl et al., 2018; Renner et al., 2019), but since the plantar surface is often not aligned to the vertical, it may be more appropriate to calibrate to the resultant.

IPSs are a promising option for wearable sensing because they are portable, cheaper than FPs, and can continuously record data (Chen et al., 2016) during daily life and not just in a laboratory setting. However, IPSs must still overcome barriers to large-scale clinical adoption such as cost, accuracy, and simplicity of calibration procedures (Schall et al., 2018). Existing commercial IPSs are expensive, costing around USD$ 10,000–20,000 (Dyer and Bamberg, 2011); however, some custom-built sensor systems have been created, costing around USD$ 150–800 (Bamberg et al., 2008; Howell et al., 2013; Jacobs and Ferris, 2015). Both commercial and custom-built systems exhibit moderate accuracy when compared against a FP standard, reporting vertical GRF to within 4–5% RMSE (Fong et al., 2008; Rouhani et al., 2010; Howell et al., 2013; Jacobs and Ferris, 2015) and maximum vertical GRF to within −1 ± 5% error (Howell et al., 2013). Furthermore, these results rely on complex calibration methods, such as artificial neural networks (Jacobs and Ferris, 2015) or universal approximators (Rouhani et al., 2010), to obtain results from multiple sensors (custom: 5–12 (Howell et al., 2013; Jacobs and Ferris, 2015); commercial Pedar insoles: 99 (Fong et al., 2008; Rouhani et al., 2010). Additionally, some IPSs have bulky and inflexible structures that can cause discomfort (Bamberg et al., 2008; Jacobs and Ferris, 2015). The novel sensors presented in this study offer advantages over prior sensors through their thin and flexible fabric-based structure and their large sensing range of 0.0025–40 MPa (Doshi and Thostenson, 2018). An IPS that uses fewer sensors, less complex calibration methods, and directly integrates sensing onto a fabric structure could be easier to implement in a clinical setting.

These desirable IPS properties could be met by applying recent advancements in nanocomposite technology that allow commonly used fabric materials to be coated in conductive carbon nanotubes (Doshi and Thostenson, 2018). This technology has already proven useful for applications such as structural health monitoring (Lim et al., 2011). Now, our research group has applied this technology to fabric-based sensors that are sensitive to pressure (Doshi et al., 2019a,b). In this study, we seek to validate a system that uses two fabric-based nanocomposite sensors to measure resultant GRF during walking.

## 2. Materials and methods

### 2.1. Overview

A complete method for obtaining peak and maximum forces from the IPS and assessing its agreement with the FP is described below. This includes the following steps: sensor fabrication and sensing mechanism; hardware design; data collection; zeroing, filtering, and resampling of data; individual sensor calibration; total IPS force calibration; gait cycle segmentation; extraction of peak and maximum forces; analysis of agreement between IPS and FP measurements; and simulation of IPS performance in distinguishing forces above and below a 50% body weight (BW) when following a PWB regimen.

### 2.2. Sensor fabrication and sensing mechanism

The sensors used in this study are developed using a novel method that gives thin and flexible fabrics pressure-sensing capacity over a wide sensing range of 0.0025–40 MPa with a gauge factor of 0.05 MPa (Doshi and Thostenson, 2018). Briefly, the process for developing these sensors consists of the following steps: An aqueous electrophoretic deposition (An et al., 2012) technique is used to deposit a thin film of carbon nanocomposite on nonwoven aramid fabrics. The process creates a thin, porous, and conformal nanocomposite film around each fiber in the nonwoven aramid fabric, making it conductive and providing sensing functionality. When the coated aramid fabric is compressed under the subject’s weight, the number of contact points between the conductive fibers increases, leading to a change in electrical resistance (Doshi and Thostenson, 2018). The change in electrical resistance is proportional to the applied pressure, which enables the measurement of GRF. Prior work from our group has demonstrated that these sensors have a large sensing range of 0.0025–40 MPa.

### 2.3. Hardware

**Figure 1.**
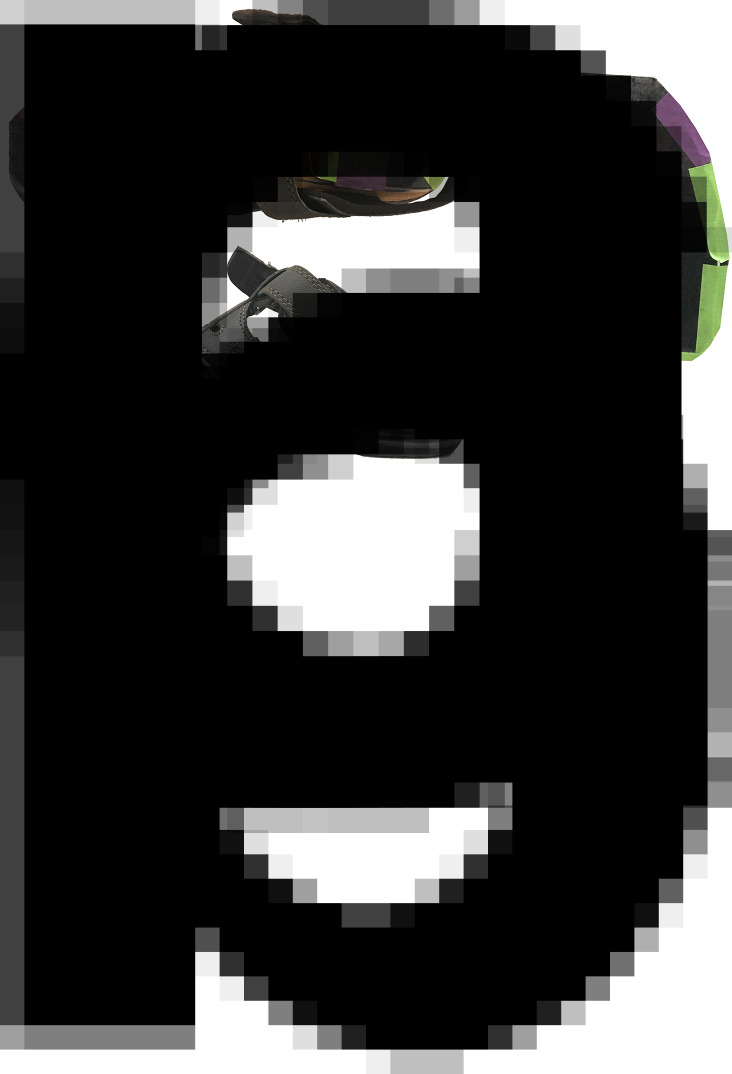
Sandal with nanocomposite sensors on the hindfoot and forefoot regions of the insole viewed from (a) above and (b) the medial side. (c) An individual nanocomposite sensor.

The wearable GRF sensor system in this study uses two sensors placed on a right-footed sandal; one located on the forefoot and the other on the hindfoot (Figure 1). Sensors were connected to an offboard circuit that extracted data using an Arduino Uno Rev3 (USD$ 23) with an external analogue-to-digital converter (USD$ 15), and data were stored in a microSD card (microSD adapter: USD$ 7.50). A voltage-dividing circuit was created to measure the change in the sensor values with compression. With sensors costing only a few dollars, the complete system costs around USD$ 50, far less than commercial systems and other custom-built systems.

### 2.4. Data collection

Ten healthy subjects (age: 23.3 ± 2.5 years; mass: 75.2 ± 18.2; five male and five female) were recruited to participate in this study approved by the University of Delaware Institutional Review Board (approval number 943311). Each subject performed calibration trials and walking trials on an instrumented treadmill (Bertec Corp, Worthington, OH). Two calibration trials were recorded, one per sensor, in which the subject repeated three “steps.” Each “step” involved slowly transitioning one’s weight onto only the hindfoot or forefoot and then back off, thus loading the sensor from 0 to BW and back to 0. Six 30-s walking trials were recorded, with two repetitions at three speeds (0.5, 1.0, and 1.5 m/s). For each of the walking trials, we instructed subjects to “stomp” on the sensor after the trial began. This practice provided a detectable event in both the IPS and FP allowed us to synchronize them. During all trials, force data were recorded by the FP at 2,000 Hz and resistance data were recorded by the Arduino at a frequency of 28 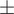 1.2 Hz.

### 2.5. Data processing

**Figure 2.**
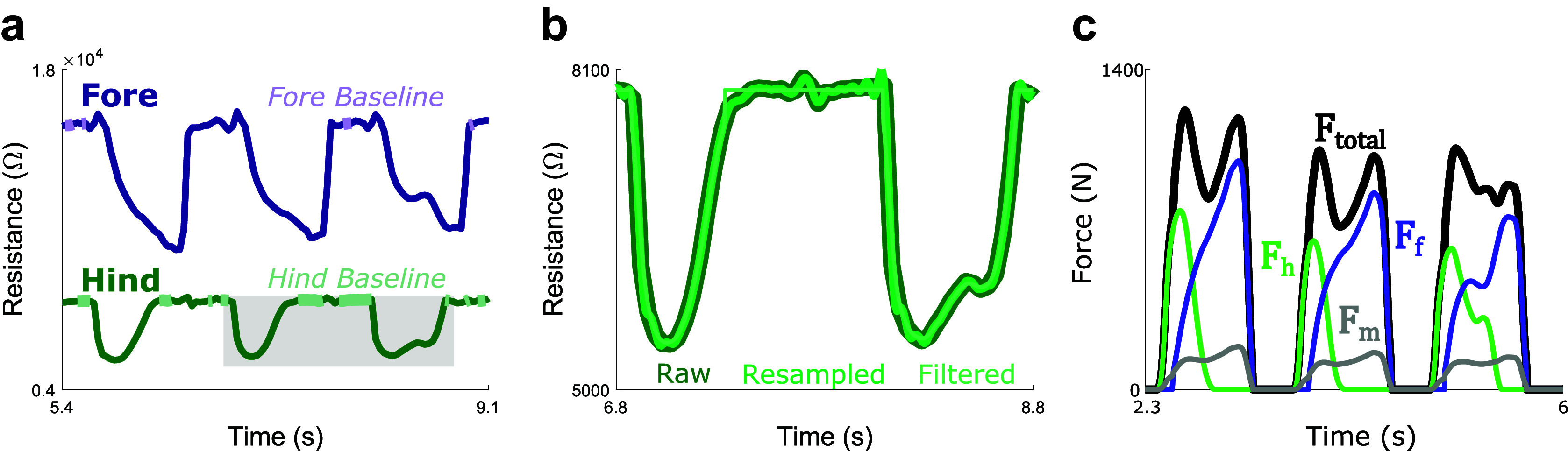
Progression of data processing from raw signals to final summed IPS force. (a) Depicts raw hindfoot (dark green) and forefoot (dark purple) resistance signals during a walking trial and the “baseline” regions (light colors); the gray box highlights the region depicted in (b). (b) Depicts raw resistance (dark green), resampled resistance (green), and final resistance (resampled, filtered, and swing phase noise removed; light green) from the hindfoot sensor. (c) Depicts the hindfoot (bright green) force signal, the forefoot (bright purple) force signal, the total force estimated with linear regression in the secondary calibration (black), and the midfoot force estimated by that calibration (gray).

GRF components recorded by the FP were resolved into resultant GRF, which was low pass filtered at 20 Hz with a fourth-order Butterworth filter. The IPS data were processed through a series of steps depicted visually in Figure 2a,b. First, IPS data were resampled in Matlab (FIR antialiasing lowpass filter with 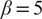 ) to a frequency of 2,000 Hz to compare it one-to-one with FP data. Then, IPS data were filtered with a binomial filter of *n* = 10,000 convolutions, which approximately functions as a Gaussian filter. To account for drift, individual sensors were zeroed for each trial by subtracting “baseline” forces recorded while the sensor was not loaded. Gait cycles were segmented using heel strike (HS) events. A FP HS was identified whenever the force first exceeded 20 N. An IPS HS was identified whenever the resistance dropped below 98% of the baseline resistance and remained below this threshold for at least 0.25 s. Finally, IPS and FP forces were normalized to BW units by dividing force by the BW for each subject.

Additionally, foot area was measured for each subject to evaluate the effect of this measure on outcomes. Area measurements were obtained by using ImageJ software to trace outlines of subjects’ feet, scanning those outlines, and then calculating the enclosed foot area.

### 2.6. Calibration

To calibrate each individual sensor, data from the IPS and FP were extracted during the three loading phases of the calibration trials, which is when the subjects shifted their weight onto the sensor. Loading phase was manually identified from initial contact of the sensor (approximately 0) up to the first peak. The MATLAB *resample* function (2 × 3 × 1 FIR antialiasing lowpass filter with 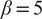 ) was used to downsample IPS resistance and FP force to correspond with uniform sampling of IPS resistance every 150 Ω. Then, the sensors were calibrated by using the least-squares method to fit a second-order polynomial that related inverse IPS resistance to FP force. The calibration was performed for each individual subject and each sensor because the sensors have a unique response due to factors such as the area of the sensor depressed by the foot, which relates to the subject’s foot size.

A second calibration was applied to the IPS system to approximate the total force on the foot during walking, in which the least-squares method was used to scale hindfoot and forefoot forces to account for forces in the unsensed midfoot region ( 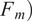 . First, FP forces ( 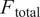 ) from the two peaks of each gait cycle were identified along with hindfoot ( 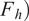 and forefoot ( 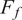 ) sensor forces at the corresponding times. Then, the least-squares method was used to determine the coefficients 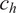 and 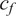 that allowed the following equation to best approximate total force as measured by the FP:
(1)

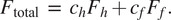

The effect of this calibration can be seen in Figure 2c, which depicts 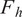 , 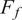 , 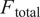 , and the estimated midfoot forces, which are calculated using equation (2):
(2)

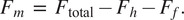



### 2.7. Data analysis

IPS validation studies have typically reported data in terms of RMSE (Fong et al., 2008; Rouhani et al., 2010; Howell et al., 2013; Jacobs and Ferris, 2015) or correlation coefficients (Fong et al., 2008; Rouhani et al., 2010; Howell et al., 2013), which only indicate how strongly two variables are related. A more appropriate method for comparing two sensors is the Bland–Altman method (Bland and Altman, 1986; Giavarina, 2015), which assesses agreement by directly comparing measurement differences. We used this method to evaluate the agreement between the IPS and FP for measuring (1) the two peaks of the GRF curve (2PK) and (2) the absolute maximum GRF (MAX) in each gait cycle. In the 2PK assessment, FP peaks were identified as follows: the maximum value in the first 30% of the gait cycle (approximately 50% of stance phase) was identified as the weight acceptance peak, and the maximum value after 30% was identified as the push-off peak. IPS peaks were identified as the two largest peaks with a width greater than 0.05 s. Corresponding peaks were compared one-to-one. In the MAX assessment, only the absolute maximum value in each gait cycle for each system was recorded, and maxima were compared one-to-one. The mean of differences (MoD) was computed to quantify bias in IPS measurements relative to FP measurements. The value which defines the distance between the MoD and the limits of agreement (abbreviated as 2S), which is equal to 1.96 standard deviations, was computed to express the range within which IPS and FP agree. Finally, least-squares linear regression was applied to the Bland–Altman results to evaluate how sensor bias varied with force magnitude.

Stratified two-fold cross-validation was implemented on an intrasubject basis to evaluate calibration performance for each individual. Stratification was used to reduce sample variance (Forman and Scholz, 2010) and bias (Picard and Cook, 1984). On each iteration, the data was split into a calibration subset and an equal-sized test subset with which the calibration was assessed. Data was stratified such that, within each iteration, both the calibration subset and the complementary test subset contained one trial from each of the three walking speeds; eight total iterations were performed to account for all possible combinations. For each of the eight iterations, all analyses from peak force calibration to the Bland–Altman analysis were carried out. The outcomes from all repetitions were compiled to quantify mean performance for outcome parameters. For each subject, a representative test subset was identified as the subset that, when defined based on MoD and 2S of the MAX assessment, exhibited the distribution that overlapped most with the mean cross-validation distribution.

### 2.8. PWB simulation

Finally, these sensors were evaluated by simulating their measurements during a clinical PWB application. The mean and standard deviation of maximum GRF (50 ± 25% BW) recorded from subjects walking under 50% PWB instructions in a prior study (Li et al., 2001) were used to generate maximum GRF values from *n* = 1 × 10^7^ gait cycles. Sensor errors were simulated for each force value assuming a normal distribution defined by the differences-versus-magnitude least-squares regression line and corresponding standard deviation about that line. Sensor measurements were computed by adding these errors to the simulated forces. Finally, the ability of this IPS system to detect overloading was evaluated by classifying forces above or below the desired 50% BW limit, and then evaluating sensitivity (the ratio of correctly identified forces below 50% BW to the true count of maximum forces below 50% BW) and specificity (the ratio of correctly identified forces above 50% BW to the true count of maximum forces above 50% BW).

## 3. Results

### 3.1. Calibration

**Figure 3.**
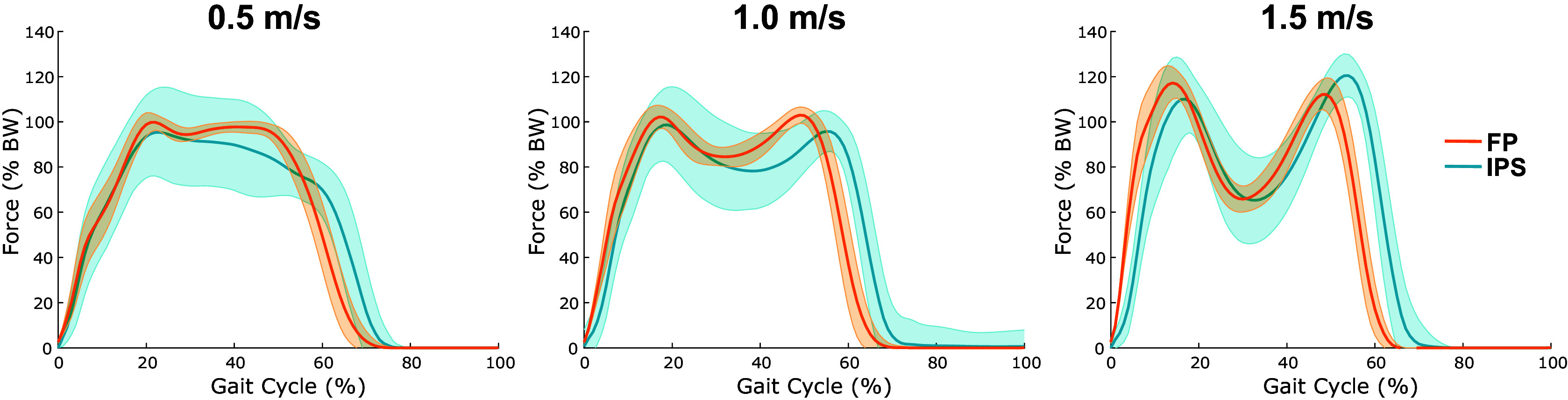
Mean resultant ground reaction force curves for both sensor systems at each speed. Each panel shows the mean GRF curves, normalized by body weight, for all seven subjects and across all gait cycles for FP (orange) and IPS (blue). Shaded error regions depict one standard deviation above and below the mean.

Results were obtained for 7 out of 10 subjects, with results from three subjects being excluded from analysis due to excessive noise in the data. The individual sensor calibrations yielded strong relationships between sensor resistance and force (Hindfoot: *r* ^2^ = 0.94 ± 0.05; Forefoot: 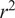 = 0.95 ± 0.03). The secondary calibration used to estimate the total force on the foot during walking produced IPS forces comparable with FP forces in both shape and magnitude (Figure 3). Like the initial calibration, this calibration returned distinct results across subjects. The hindfoot coefficient varied between 0.69 and 2.33 and the forefoot coefficient varied between 0.71 and 1.38.

### 3.2. Bland–Altman analysis

**Figure 4.**
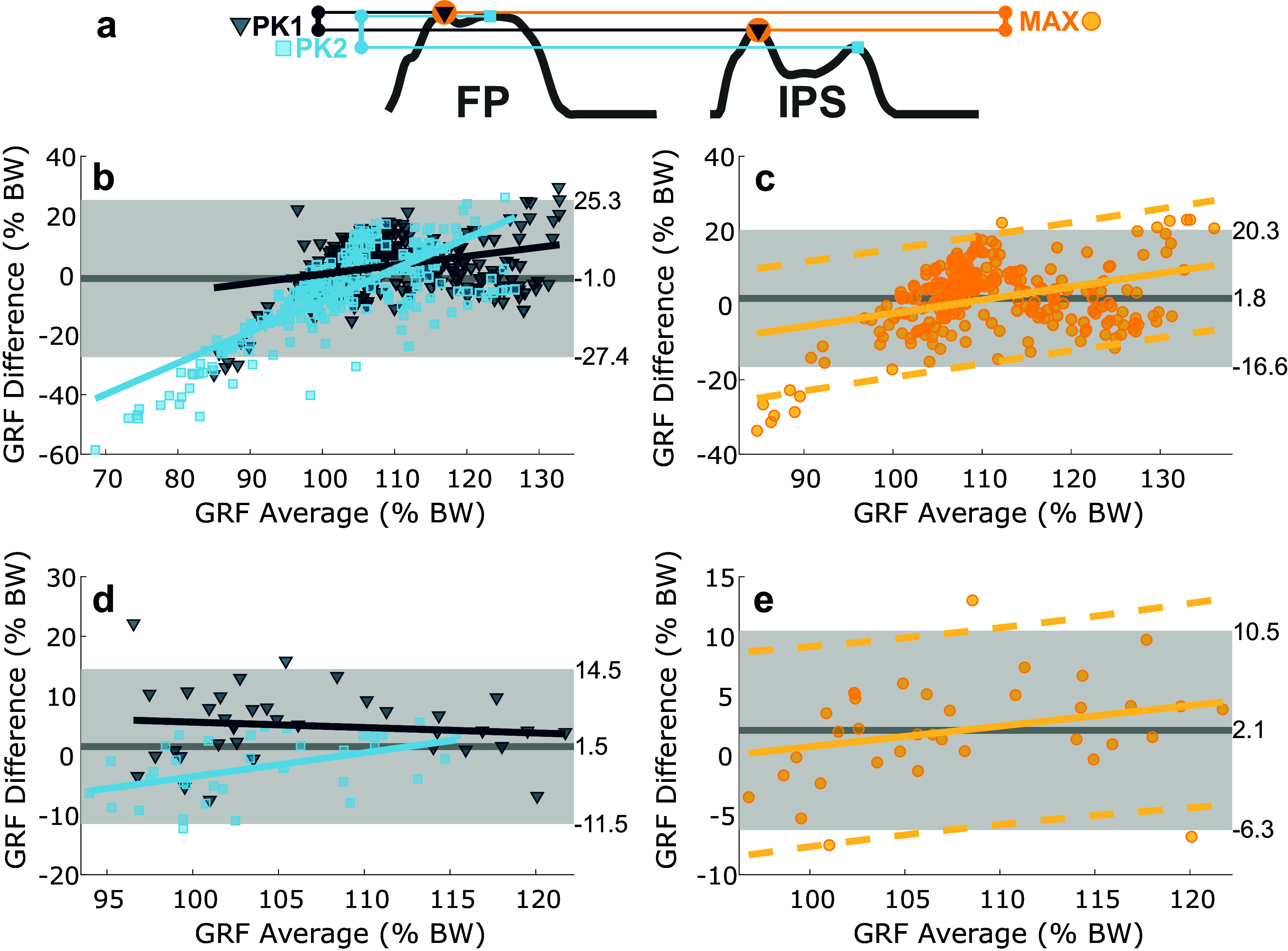
Comparison of FP and IPS systems under both 2PK and MAX assessments. (a) Depicts the relevant forces identified from each system within one gait cycle. Bland–Altman plots are depicted as (b) group results for 2PK with first peaks (PK1) in dark blue and second peaks (PK2) in light blue (PK1 regression line: *y* = 0.30*x* − 30% BW; PK2 regression line: *y* = 1.05*x* − 113% BW), (c) group results for MAX (regression line: 0.36*x* − 38% BW), (d) representative subject results for 2PK assessment (PK1 regression line: −0.09*x* + 15% BW; PK2 regression line: 0.40*x* − 44% BW), and (e) representative subject results for MAX assessment (regression line: 0.17*x* − 16% BW). In all plots, solid gray lines depict the MoD and gray regions indicate 95% confidence interval defined by the limits of agreement. In the 2PK plots, the MoD and limits of agreement are determined by both the first and second peaks. Furthermore, dark blue points represent the first peaks and light blue points represent the second peaks. The corresponding colored lines are linear regression lines for the respective peaks. In MAX plots, the solid orange line is a linear regression line and dashed orange lines depict the upper and lower bounds of the 95% confidence intervals about that line.

The IPS and FP exhibited comparable results under both the 2PK and MAX assessments (Figure 4 and Table 1). Slight, but significant, bias was observed in both assessments. Under the 2PK assessment, the MoD was typically negative, with a statistically significant negative bias identified in five of seven subjects and in the group. However, the bias was small, with five subjects having MoD with absolute values less than 1.5% BW (Table 1). Under the MAX assessment, the MoD was typically positive, with a statistically significant positive bias identified in six of seven subjects and in the group. Again, the bias was small, with five subjects having MoD with absolute values less than 2.5% BW (Table 1). 2S values varied more substantially across subjects (Figure 4 and Table 1), with values of 25.4 ± 11.1% BW for the 2PK assessment and 15.8 ± 9.3% BW for the MAX assessment.

**Table 1.** Foot areas, MoD, and 2S for each subject and the group mean

		2PK		MAX	
Subject	Foot area (cm^2^)	MoD (% BW)	2S (% BW)	MoD (% BW)	2S (% BW)
1	39.2	−1.5 ± 0.8	22.9 ± 0.8	0.7 ± 0.9^a^	9.6 ± 0.7
2	30.6	−0.7 ± 1	22.2 ± 0.8	1.3 ± 1^a^	19.4 ± 0.9
3	23.4	−8.9 ± 0.7	44.2 ± 0.7	−2.4 ± 1.1	35.3 ± 0.8
4	34.2	−0.2 ± 0.5	21.8 ± 0.8	0.8 ± 0.8^a^	12.5 ± 0.4
5	33.7	4.9 ± 3.1^a^	17 ± 1.1	7.3 ± 3.2^a^	12.1 ± 2.3
6	33.5	1.1 ± 1.3^a^	13 ± 1.5	2.1 ± 1.4^a^	8.5 ± 0.7
7	41.7	−1 ± 2.0	27.1 ± 1.4	5.8 ± 2.6^a^	11.6 ± 1.2
Mean	33.7 ± 5.9	−1.3 ± 4.3	25.4 ± 11.1	1.9 ± 3.0	15.8 ± 9.3

*Note.* Each value is reported as mean ± standard deviation, which is obtained from the eight cross-validation iterations.

a Indicates a significant difference (*p* < .05) between MoD and zero.

There was a slight measurement bias in all subjects that increased as the magnitude of the measurement increased (Figure 4c,d). This upward trend in bias was especially apparent in subjects that had worse (higher) 2S values. Small foot areas led to a decreased agreement between IPS and FP. Subjects with foot areas smaller than 33 cm^2^ exhibited 2S values in excess of 19% BW for the MAX assessment, while subjects with large feet exhibited 2S values between 8 and 13% BW (Table 1).

### 3.3. Simulation of PWB application

The simulation of a PWB application demonstrated good classification metrics for the identification of overloading. Under the 50% PWB condition, the simulation predicted good specificity (0.83 ± 0.08) and good sensitivity (0.85 ± 0.25) (Table 2).

**Table 2.** Sensitivity and specificity results from simulations of a 50% PWB regimen

Subject	1	2	3	4	5	6	7	Mean
Sensitivity	0.992	0.815	0.308	0.906	1.000	0.952	1.000	0.85 ± 0.25
Specificity	0.862	0.854	0.824	0.909	0.694	0.913	0.753	0.83 ± 0.08

## 4. Discussion

This study introduced novel fabric-based pressure sensors for GRF measurement and demonstrated accurate force measurements across a range of walking speeds. Individual sensor calibration yielded strong relationships between sensor resistance and force. The secondary total force calibration allowed the IPS to account for unsensed foot regions and obtain better overall force estimates.

With subjects exhibiting MoD of 1.9 ± 3.0% BW (18 ± 17 N) and 2S of 15.8 ± 9.3% BW (118 ± 61 N), our results reflect greater agreement than prior studies that exhibited MoD of −48 N and 2S of 164 N for running at 2.2–3.3 m/s (Seiberl et al., 2018) and MoD of 36 N and 2S of 200 N for walking at 1.0 and 1.7 m/s (Braun et al., 2015). Likewise, a commercial Novel loadsol IPS exhibited comparable-to-worse agreement when compared with our study, with one study reporting MoD of −40.1 N and 2S of 119.7 N for walking at 1.4 m/s (Burns et al., 2019), and another studying reporting MoD of 2% BW and 2S of approximately 30% BW (Renner et al., 2019) for walking at 1.3 m/s. However, it is worth noting that our method required the use of a FP for calibration while the loadsol is calibrated using only a BW measurement (Burns et al., 2019). The use of a FP would likely give our system an advantage in terms of measurement accuracy since more data points are collected.

One advantage of our calibration method compared to other studies was that our results were obtained by using simple least-squares regression to calibrate individual sensors and to compute the total force as a sum of scaled sensor forces. Other studies have used machine learning methods to optimize sensor calibration (Rouhani et al., 2010; Jacobs and Ferris, 2015). Our method offers two advantages over this approach. First, our method is less computationally complex and thus can be performed more rapidly. Second, our method provides a more simple and realistic mapping of measured signals to total GRF: the total GRF is simply treated as a scaled sum of individually measured forces to account for regions not measured.

The clinical value of this system versus the commercial loadsol can be evaluated according to the criterion for biofeedback in PWB proposed by van Lieshout et al. (2016), which requires the lower limit of agreement to be less than the prescribed range of PWB forces. Our study exhibited a lower LoA of −16.6 %BW, and so it likely would be suitable for PWB requirements in all three ranges (1–20, 20–50, and 50–75 %BW) evaluated in van Lieshout et al. (2016). However, the loadsol would likely only meet the criterion for the 20–50 %BW range given its lower LoA of approximately 30 %BW. Consequently, our IPS would allow for a more precisely graded progression of PWB. This advantage could be important for applications such as patients recovering from autologous chondrocyte implantation, where a more graded progression would steadily increase limb loading to promote bone remodeling without overloading the recovering bone structure (Ebert et al., 2008).

This sensor system offers value by achieving these results with a cheaper and simpler system that can be used to obtain portable measurements. Furthermore, we identified a feasible application for this sensor system by simulating its ability to detect overloading during PWB. Given the sensitivity and specificity outcomes, we expect that these sensors could reliably discriminate loads exceeding PWB thresholds from those within PWB thresholds, and therefore provide a valuable feedback system to help monitor and train patients to comply with PWB instructions.

For both the 2PK and MAX assessments, MoD values were low, indicating that multi-step averaged values from the IPS offer reliable accuracy. However, the method for handling the data altered the reliability of sensor measurements. Measurements were more precise under the MAX assessment than the 2PK assessment. This outcome is reasonable since the MAX assessment only considers the magnitude of these maxima in IPS and FP but does not account for whether or not they occur at the same peak.

The reliability of measurements for most subjects demonstrates that, within a certain range of foot sizes, this generic sensor system can be calibrated to return accurate force estimates. However, smaller foot sizes under 33 cm^2^ are too small for this IPS to obtain accurate measurements. Another pair of sensors integrated with smaller footwear could potentially work better for individuals with smaller foot sizes.

This study had a few limitations related to design of the sensor system. First, three subjects had to be excluded from data analysis due to excessive noise in the data, which may have been caused by degradation of the connection between sensor and wires after repeated use. This connection consists of a conductive silver paint (SPI supplies) and a 2-part conductive epoxy resin (Epoxies 40–3900), and the resin could wear away with repeated use. In the future, this problem can be avoided by placing these connections on regions of the sensor subject to less external forces. Additionally, the use of only two sensors to estimate the total force on the foot necessitated a secondary calibration to account for unmeasured forces in the midfoot. Drift and hysteresis introduced uncertainty in individual sensor measurements. The IPS sampling frequency was below the 100 Hz recommendation of Pedobarographic Group of the International Foot and Ankle Biomechanics (iFAB) (Giacomozzi et al., 2012). This limitation reduced our confidence in quantifying continuous GRF metrics, and so our analysis focused instead on point measurements, namely MAX and 2PK measurements. Although the iFAB standard was not achieved, it is worth noting that the iFAB statement indicates that there is “possibility to work at lower rates” (Giacomozzi et al., 2012) and, furthermore, that the frequency of the GRF waveform predominantly lies in the range of 10 Hz, except during HS transients (Simon et al., 1981). When measuring a 10 Hz frequency, our sampling frequency of 28 Hz meets the Nyquist–Shannon sampling theorem since it is more than double the measured frequency. Furthermore, the results of the study show that our IPS was suitable for the application since the maximum forces measured by the IPS exhibited comparable-to-greater agreement with FP measurements than prior studies (Braun et al., 2015; Seiberl et al., 2018; Burns et al., 2019; Renner et al., 2019) and the PWB simulations indicated strong classification metrics for identification of overloading. We believe these results indicate the potential of these sensors to provide reliable wearable measurements for applications such as biofeedback during PWB. Future work on this system will involve updating the data acquisition system to increase the sampling frequency.

There were also limitations related to calibration. Individual sensors were only calibrated up to BW, which means that larger forces require extrapolation beyond the calibration range. The lack of a model to relate resistance to force could have led to systematic errors that vary in force magnitude. Adjustments to strap tightness increased compression between foot and sandal, leading to uncertainty in baseline forces; this issue is especially problematic when drift is considered, since the effects of these two phenomena on baseline resistance cannot be isolated. Manual alignment of datasets introduces the potential for error and bias. Additionally, there appeared to be a slight shift in toe-off and peak timings (Figure 3). This phenomenon may be due to data processing errors in identifying pressure application and release timing on each sensor or due to actual physical deformation of the sensors after contact with the ground is lost. Finally, this IPS system was only calibrated for walking, which means that the measurements may not generalize well to other activities. Future work could be done to improve the design of this IPS system, such as building the sensors into insoles, increasing sampling frequency, and adding a sensor in the midfoot region.

The sensor system in this study offered accurate force measurements comparable to prior IPS studies but with a simpler, low-cost system. By evaluating the differences relative to FP measurements, we quantified the limits of agreement and measurement bias to understand the range of this sensor’s validity. Then, the sensor’s performance in a PWB regimen was simulated to demonstrate its potential for this application. Given the validity exhibited by this simple and affordable system, we anticipate that this sensor technology has the potential to overcome typical barriers to widespread usage of wearable insole force sensors and make real-world GRF measurements more common in clinical practice.

## Data Availability

Data available upon request – please contact Kaleb Burch at kburch@udel.edu or Jill Higginson at higginso@udel.edu.
